# Mechanism of action of synaptic mitochondrial damage in delayed cognitive recovery

**DOI:** 10.4103/NRR.NRR-D-24-01126

**Published:** 2025-03-25

**Authors:** Huihui Miao, Qiang Liu, Yan-Ping Liu, Bin-Bin Yan, Xin-Hao Jiao, Hai-Bi Wang, Cheng-Hua Zhou, Tianzuo Li, Zhongcong Xie, Yuqing Wu

**Affiliations:** 1 Department of Anesthesiology, Beijing Shijitan Hospital, Capital Medical University, Beijing, China; 2 Department of Anesthesiology and Perioperative Medicine, Shanghai Fourth People’s Hospital, School of Medicine, Tongji University, Shanghai, China; 3 Jiangsu Province Key Laboratory of Anesthesiology/NMPA Key Laboratory for Research and Evaluation of Narcotic and Psychotropic Drugs, Xuzhou Medical University, Xuzhou, Jiangsu Province, China; 4 Jiangsu Key Laboratory of New Drug Research and Clinical Pharmacy, Xuzhou Medical University, Xuzhou, Jiangsu Province, China; 5 Department of Anaesthesia, Critical Care and Pain Medicine, Massachusetts General Hospital and Harvard Medical School, Charlestown, MA, USA

**Keywords:** acetylase, aged mice, cyclophilin D, delayed neurocognitive recovery, hippocampus, long-term potentiation, mitochondrial permeability transition pore, sirtuin 3, synaptic mitochondria, synaptosome

## Abstract

Delayed neurocognitive recovery following anesthesia and surgery is a common complication in older adult patients. Synapses are fundamental to cognitive function. The activity of synapses heavily depends on the energy supplied by synaptic mitochondria, which are significantly influenced by oxidative stress. Sirtuin 3 is a histone deacetylase located in the mitochondrial matrix that plays a pivotal role in regulating mitochondrial function. However, it remains unclear whether and how sirtuin 3 is involved in the development of delayed cognitive recovery. Therefore, in this study, we investigated the potential role of sirtuin 3 in synapses during delayed neurocognitive recovery. Our results showed that anesthesia and surgery induced cognitive impairment in mice and reduced sirtuin 3 protein expression. Overexpression of sirtuin 3 inhibited opening of the mitochondrial permeability transition pore by reducing acetylation of K166 on cyclophilin D and also rescued cognitive impairment. Aged mice carrying the cyclophilin D-K166R mutation exhibited significantly reduced cognitive impairment. Similarly, administering the mitochondrial permeability transition pore blocker, cyclosporine A, effectively alleviated the decline in synaptic mitochondrial function and cognitive impairment caused by anesthesia and surgery in aged mice. These results indicate that the sirtuin 3/cyclophilin D-K166/mPTP signaling pathway in hippocampal synaptic mitochondria is involved in delayed neurocognitive recovery of aged mice, suggesting this pathway could serve as a potential target for treatment.

## Graphical abstract

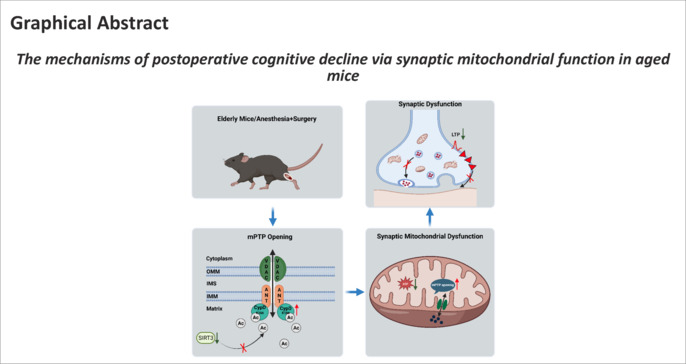

## Introduction

Delayed neurocognitive recovery (dNCR), classified as a perioperative neurocognitive disorder (Evered et al., 2018), represents a significant challenge among elderly patients within thirty days post-anesthesia/surgery. It is marked by deficiencies in memory, perception, information processing, and social integration (Hanning, 2005; Deiner and Silverstein, 2009), and potentially results in diminished quality of life, extended hospital stays, and elevated mortality rates (Steinmetz et al., 2009; Rundshagen, 2014). However, the precise pathogenesis and underlying mechanisms of dNCR remain largely unresolved.

The human brain commands roughly 20% of the body’s total energy demand (Magistretti and Allaman, 2018), with mitochondria playing a central role in this energy supply within the brain. Specifically, mitochondria predominantly generate adenosine triphosphate (ATP) through oxidative phosphorylation. The energy-intensive processes of synaptic signaling transmission and neurotransmitter transport impose substantial energy needs (Cunnane et al., 2020; Li and Sheng, 2022). Notably, synaptic mitochondria, which accumulate in great numbers at synapses, exhibit distinctive attributes in terms of size, activity, and lifespan compared with non-synaptic mitochondria (Li et al., 2004; Verstreken et al., 2005; Gao et al., 2022). Additionally, with increasing age, synaptic mitochondria are more vulnerable to diverse stressors in contrast to their non-synaptic counterparts (Martinez et al., 1996; Banaclocha et al., 1997). Alzheimer’s disease (AD) transgenic mice have shown premature degradation of synaptic mitochondria, resulting in compromised aerobic respiration and abnormal peroxide accumulation (Du et al., 2010). Furthermore, a significant decline in oxidative damage and activity of the antioxidant enzyme superoxide dismutase in the hippocampus of aged rats has been demonstrated following anesthesia and surgery (Netto et al., 2018). Intriguingly, enhancing mitochondrial biogenesis and diminishing mitochondrial oxidative damage can reverse cognitive and behavioral deterioration in AD transgenic mice (Zhang et al., 2015). In light of these insights, we sought to determine whether enhancing energy supply at synapses via modulation of synaptic mitochondrial function could emerge as a potential therapeutic avenue for addressing dNCR.

The pivotal role of the mitochondrial permeability transition pore (mPTP) in maintaining mitochondrial integrity is well-established (Morciano et al., 2021; Shi et al., 2021). This structure comprises essential components, including a voltage-dependent ion channel, adenine nucleotide translocator, and cyclophilin D (CypD) (Baines et al., 2005; Zhang et al., 2023). Remarkably, CypD knockout mice have shown resistance to various neurodegenerative disorders (Forte et al., 2007; Martin et al., 2009). Particularly notable is the response of CypD to heightened oxidative stress, triggering a significant increase in acetylation levels of CypD. Acetylated CypD then binds to adenine nucleotide translocator, causing the formation of a tunnel-like structure bridging the mitochondrial matrix and cytoplasmic solutes. The opening of this tunnel-like conduit induces the electrochemical transfer of numerous ions and solutes from the extramitochondrial environment into the mitochondrial matrix. This process culminates in attenuation of the mitochondrial membrane potential (MMP), ensuing mitochondrial swelling, and a diminution in energy supply (Hafner et al., 2010). Despite this understanding, the precise role of acetylated CypD in the context of synaptic mitochondria during the course of dNCR remains enigmatic.

Sirtuin 3 (SIRT3), a nicotinamide adenine dinucleotide-dependent deacetylase, primarily resides within mitochondria and is the solitary sirtuin recognized for its contribution to human lifespan extension (Cheng et al., 2016; Diao et al., 2021; Zhang et al., 2022). SIRT3 exerts deacetylation control over mitochondrial function and is notably linked to an array of age-associated ailments, encompassing cardiovascular and neurodegenerative conditions (Hirschey et al., 2010). The absence of SIRT3 in knockout mice has been correlated with marked impairments in learning, memory, and long-term potentiation (LTP) (Kim et al., 2019). Interestingly, a study found that SIRT3 overexpression yields a reduction in CypD acetylation levels, potentially mitigating cognitive impairment in mice (Sun et al., 2017a). Furthermore, within an aging neurodegenerative mouse model, SIRT3 was shown to directly deacetylate CypD at the K166 site (Hafner et al., 2010). Another study reported a pathogenic role of SIRT3/CypD acetylation in endothelial dysfunction and hypertension (Dikalova et al., 2024). Nevertheless, more comprehensive investigation is needed to determine whether the involvement of SIRT3 in the development of dNCR is related to deacetylation of CypD-K166, and subsequently, bolstering of synaptic mitochondrial function.

Therefore, our hypothesis in the present study was that SIRT3-mediated CypD-K166 deacetylation regulates synaptic mitochondrial function within the context of anesthesia/surgery-induced cognitive impairment among aged mice. This study will help understanding on the pathogenesis of perioperative neurocognitive disorders and their therapeutic interventions.

## Methods

### Animals

Aged male C57BL/6 mice (18 months old; weight 30–35 g) were procured from the Animal Center of Xuzhou Medical University, China (animal license No. SYXK-2020-0048). B6/JNju-Ppif em1Cin (K166R)/Gpt mutant mice (simplified as CypD-K166R mutant mice; strain No. T010902; MGI: 7304562) were purchased from Gempharmatech Co., Ltd. (Nanjing, China). The mice were group-housed in a vivarium under controlled conditions (temperature: 22–25°C, 12-hour light/dark cycle) with four or five mice per cage. Mice were provided with food and water *ad libitum*. All animal experiments were approved by the Ethics Committee on Experimental Animals of Xuzhou Medical University (approval No. 201910A033) and were conducted in compliance with the Animal Research Reporting *In Vivo* Experiments (ARRIVE) guidelines (Percie du Sert et al., 2020) and the National Institutes of Health Guide for the Care and Use of Laboratory Animals.

### Establishment of a delayed neurocognitive recovery model

To establish a dNCR model, mice underwent intramedullary fixation of tibial fractures following a previously described procedure (Zhou et al., 2023). Briefly, mice were anesthetized with 2.0% isoflurane (RWD Life Science Co., Ltd., Shenzhen, China), and a skin incision was introduced below the knee joint to expose the tibia. A 0.4-mm stainless steel needle was inserted into the intramedullary cavity of the tibia at the tibial tuberosity, and the middle of the tibia was fractured by clamping it with surgical forceps. The entire surgical procedure, from induction of anesthesia to end of surgery, lasted approximately 20 minutes. During surgery, the body temperature of mice was maintained between 36°C and 37°C using a heating pad. After surgery, 2% lidocaine ointment was applied to the wound suture to alleviate postoperative pain. The surgical procedures were performed aseptically, and mice were returned to their cages for recovery after awakening.

### Grouping and treatment

**Figure 1:**
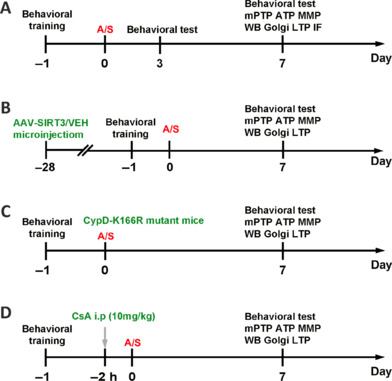
Schematic diagram of the experimental procedure. (A) The mice received behavioral training on day 1 prior to A/S and behavioral tests on days 3 and 7 after A/S. A/S was performed on day 0. Hippocampal tissues were immediately harvested at the end of behavioral testing. Western blotting of SIRT3, synaptophysin, and PSD95 expression was performed. Levels of mPTP opening, MMP, and ATP were measured on day 7. Golgi staining and LTP of synaptic function were measured on postoperative day 7. (B) AAV-SIRT3/VEH virus was microinjected into the CA1 region of the hippocampus 28 days before A/S. The mice received behavioral training at day –1 and behavioral tests on days 7 after A/S. Brain tissues were immediately harvested for biochemical assays at the end of behavioral testing. (C) CypD-K166R mutant mice were used for this experiment. The mice received behavioral training on day –1 and behavioral tests on day 7 after A/S. Brain tissues were immediately harvested for biochemical assays at the end of behavioral testing. (D) Behavioral training was performed 1 day prior to A/S, and CsA was administered 2 hours before A/S. After behavioral testing, mice were sacrificed for biochemical assays. A/S: Anesthesia/surgery; ATP: adenosine triphosphate; CsA: cyclosporine A; IF: immunofluorescence; i.p: intraperitoneal; LTP: long-term potentiation; MMP: mitochondrial membrane potential; mPTP: mitochondrial permeability transition pore; SIRT3: sirtuin 3; VEH: vehicle; WB: western blot.

Tibial fracture procedures were performed under isoflurane anesthesia to establish the dNCR model. In the first experiment, the mice were randomly placed into two groups: a control group (C) and anesthesia/surgery group (A/S). The mice in group C received no anesthesia/surgery procedure. For the anesthesia/surgery group, mice received 2.0% isoflurane for maintenance with surgery. In the second experiment, the mice were randomly divided into four groups: a control + control adeno-associated virus (AAV) vehicle (VEH) group (C + VEH), anesthesia/surgery + control AAV-VEH group (A/S + VEH), control + AAV-SIRT3 vector group (C + SIRT3), and anesthesia/surgery + AAV-SIRT3 vector group (A/S + SIRT3). In the third experiment, the mice were randomly divided into four groups: wild-type mice with control condition group (C + WT), wild-type mice with anesthesia/surgery condition group (A/S + WT), CypD-K166R mice with control condition group (C + CypD-K166R), and CypD-K166R mice with anesthesia/surgery condition group (A/S + CypD-K166R). In the last experiment, the mice were randomly divided into four groups: control mice with 1% dimethyl sulfoxide (DMSO, solvent of cyclosporine A [CsA], 0.2 mL) injection group (C + DMSO), anesthesia/surgery mice with DMSO injection group (A/S + DMSO), control mice with CsA injection group (C + CsA), and anesthesia/surgery mice with CsA injection group (A/S + CsA). A schematic diagram of the experimental procedure is shown in **Figure 1**.

### Stereotaxic surgery and virus injection

To determine the function of SIRT3 in dNCR, AAV-SIRT3 vector was administered into the dorsal hippocampal CA1 region of aged mice four weeks before anesthesia/surgery. Mice were anesthetized using 1% pentobarbital sodium (Xinyuhong Biomedical Technology Co., Ltd., Wuhan, Hubei, China) and placed on a brain stereotactic instrument (RWD Life Science Co., Ltd., Shenzhen, China). The parietal area of the mouse head was disinfected with iodophor, and a 1-cm incision was made to fully expose the skull. Burr holes were drilled above the hippocampal CA1 region for virus injection. AAV-SYN-SIRT3-mNeonGreen (200 nL virus, 100 nL/min, titers: 1.0 × 10^12^ vg/mL, OBIO Technology, Shanghai, China) was injected into the hippocampal CA1 region using an automatic microinjection pump (KD Scientific, Holliston, MA, USA). The injection needle was left in place for 10 minutes to minimize reflux. The coordinates for viral injection into the hippocampal CA1 region were: anterior–posterior (AP), –1.94 mm; medial–lateral (ML), ±1.4 mm; and dorsal–ventral (DV), –1.45 mm. Mice injected with the virus were allowed to recover for at least 3 weeks before undergoing tibial fracture surgery. Viral expression was histologically confirmed in mice at the end of the experiment.

### Drug administration

CsA (Sigma–Aldrich, St. Louis, MO, USA, Cat# SML1018) was administrated to inhibit mPTP opening. CsA was intraperitoneally injected into mice at a dose of 10 mg/kg, 2 hours before surgery.

### Open field test

The open field test (OFT) was used to examine the locomotor activity of mice at 1 day before surgery and days 3 and 7 after surgery, according to our previous study (Liu et al., 2021). Mice were group-housed in the OFT. Mice were placed in a square white plastic open-air device (50 cm long, 50 cm wide, and 40 cm tall), and their locomotor behavior was recorded with a video camera controlled using ANY-maze video tracking software (ANY-maze, Stoelting Co., Wood Dale, IL, USA). Mice were allowed to acclimate for 5 minutes and then walking distance was recorded between 6–10 minutes. After each experiment, the device was cleaned with 75% ethanol to avoid olfactory cues.

### Fear conditioning test

The fear conditioning test (FCT) was conducted as previously described (Sun et al., 2017b). Mice were group-housed in the OFT. The FCT comprised a training phase before surgery and an evaluation phase on days 3 and 7 post-surgery. The FCT was performed 30 minutes after the OFT test. One day before surgery, mice were trained in chambers to establish long-term memory, and their behavior monitored with a video camera. Each mouse was allowed to familiarize itself with the conditioning chamber for 2 minutes, followed by six pairs of conditional–unconditional stimuli. Each pair consisted of a 20-second, 70-dB tone (conditional stimulus), a 25-second contextual interval, and a 0.70-mA electrical foot shock for 2 seconds (unconditional stimulus). Each pair of conditional–unconditional stimuli was separated by a random interval ranging from 45–60 seconds. The mice were allowed to recover in the conditioning chamber for 60 seconds.

The testing phase of the FCT, which consisted of a context test and a tone test, was performed on days 3 and 7 post-surgery. For the context test, mice were placed back into the conditioning chamber for 5 minutes without a tone or foot shock. The tone test was performed 2 hours after the context test. The mice were placed in a new chamber with a different environment for 5 minutes, during which the tone was delivered for 3 minutes. The activity of mice in the chamber was recorded and freezing behavior (defined as periods in which the mouse exhibited no movements except for respiration) was automatically analyzed using a FCS camera and Med Associates Animal Behavior Analysis System (Med Associates, Inc., Fairfax, VA, USA), respectively.

### Synaptosome preparation

Synaptosomes were isolated from hippocampal homogenates using a discontinuous Percoll gradient procedure, as previously described but with slight modifications (Dunkley et al., 2008). Briefly, mice were anesthetized with pentobarbital sodium (40 mg/kg) and subsequently decapitated and executed. Fresh hippocampal tissues were extracted and homogenized using a 7-mL Dounce glass homogenizer in ice-cold preparation buffer (0.32 M sucrose, 1 mM ethylene diamine tetraacetic acid, 5 mM Tris, pH = 7.4) containing a protease inhibitor cocktail (Roche, Basel, Switzerland). The homogenate was centrifuged at 1000 × *g* for 10 minutes at 4°C to remove cellular debris and nuclei. The supernatant was carefully collected and added to a discontinuous Percoll gradient (comprising layers of 3% [vol/vol], 10% [vol/vol], 23% [vol/vol], and 40% [vol/vol] Percoll) and centrifuged at 31,000 × *g* for 5 minutes at 4°C. Synaptosomes were collected from the 10/23% interface. Collected samples were subsequently diluted with 10× volume of preparation buffer and centrifuged at 20,000 × *g* for 30 minutes at 4°C to remove excess Percoll pellets. The resulting pellet was resuspended in an isotonic physiological buffer for subsequent western blotting, MMP, and ATP analysis.

### Western blotting

Western blotting was performed as previously described (Liu et al., 2021). Briefly, synaptosomes obtained from hippocampal tissue were homogenized using ice-cold radioimmunoprecipitation assay lysis buffer (Beyotime, Shanghai, China, P0013B) containing protease and phosphatase inhibitors. Lysates were centrifuged at 13,500 × *g* for 15 minutes at 4°C to remove precipitates, and the supernatants collected. A bicinchoninic acid protein assay kit (Beyotime, P0010S) was used to determine protein concentration. Sodium dodecyl sulphate–polyacrylamide gel electrophoresis (10%–15%) was used to separate proteins (30 µg per lane), which were subsequently transferred onto poly(vinylidene fluoride) membranes (Millipore, Burlington, MA, USA). The membranes were incubated overnight at 4°C with primary antibodies, including rabbit anti-SIRT3 (1:1000, Cell Signaling Technology, Danvers, MA, USA, Cat# 5490S, RRID: AB_10828246), rabbit anti-acetylation (Ac)-CypD-K166 (1:500, ABclonal, Wuhan, China, Cat# WG-00545P), mouse anti-CypD (1:1000, Abcam, Cambridge, UK, Cat# ab110324, RRID: AB_10864110), rabbit anti-synaptophysin (1:10,000, Abcam, Cat# ab32127, RRID: AB_2286949), rabbit anti-postsynaptic density protein (PSD95) (1:1000, Cell Signaling Technology, Cat# 2507S, RRID: AB_561221), and mouse anti-beta-actin (ACTB) (1:2000, ABclonal, Cat# AC004, RRID: AB_2737399). After washing three times, the membranes were incubated with horseradish peroxidase-conjugated secondary antibodies (goat anti-mouse IgG, 1:2000, Abcam, Cat# ab6789, RRID: AB_955439; or goat anti-rabbit IgG, 1:2000, Abcam, Cat# ab97051, RRID: AB_10679369) for 1 hour at 37°C. Specific immunoreactivity was detected by enhanced chemiluminescence (Beyotime, P0018S), and the band intensity analyzed using ImageJ software (version 1.52d; National Institutes of Health, Bethesda, MD, USA) (Schneider et al., 2012).

### Co-immunoprecipitation

Synaptosomes extracted from hippocampal tissue were lysed by ultrasonication and suspended in a low concentration of immunoprecipitation buffer (20 mM Tris, 150 mM NaCl, 1% Triton X-100, sodium pyrophosphate, β-glycerophosphate, ethylene diamine tetraacetic acid, sodium orthovanadate, and leupeptin). Total protein extract was incubated with 2 μg of SIRT3 or CypD antibody (same antibodies as western blotting) and shaken overnight at 4°C. The following day, 50 μL of fully suspended protein A/G agarose was added to the antigen–antibody reaction system and shaken slowly at 4°C for 3 hours. The vial was centrifuged at 2800 × *g* for 5 minutes, and the immune precipitate collected and washed three time in low-intensity lysis buffer. The immune complex was finally resuspended in SDS–PAGE buffer for subsequent immunoblotting analysis.

### Immunohistochemistry

Mice were deeply anesthetized with pentobarbital sodium and immediately subjected to cardiac perfusion with 0.9% normal saline and 4% paraformaldehyde. Brains were removed, post-fixed in paraformaldehyde overnight at 4°C, and cryoprotected in 30% sucrose solution for 72 hours. Sections (30 µm-thick) were prepared using a freezing microtome (VT1000S, Leica Microsystems, Wetzlar, Germany). For double immunofluorescence, sections were incubated with a mixture of rabbit anti-SIRT3 conjugated to fluorescein isothiocyanate (FITC) (1:100, BiorByt, Cambridge, UK, Cat# orb103490; RRID: AB_3665898) and mouse anti-NeuN antibody (1:400, Abcam, Cat# ab104224, RRID: AB_10711040)/mouse anti-glial fibrillary acidic protein (GFAP) antibody (1:300, Cell Signaling Technology, Cat# 3670, RRID: AB_561049)/goat anti-ionized calcium binding adaptor molecule 1 (Iba1) antibody (1:200, Abcam, Cat# ab5076, RRID: AB_2224402). The secondary antibodies used were: goat anti-mouse Alexa 594 (1:400, Abcam, Cat# ab150116, RRID: AB_2650601) and donkey anti-goat Alexa 594 (1:400, Abcam, Cat# ab150132, RRID: AB_2810222). To exclude nonspecific staining of false positives, negative controls were included. Specifically, as a secondary antibody only control, phosphate buffered saline (PBS) was used instead of primary antibody. The secondary antibody was used goat anti-rabbit IgG (Alexa Fluor® 488, 1:400, Abcam, Cat# ab150077, RRID: AB_2630356) at a dilution of 1:400. After staining, sections were mounted onto glass slides, and images (20×) were obtained using a confocal microscope (Olympus, FV1000, Tokyo, Japan).

### Mitochondrial permeability transiyion pore opening assay

Fresh hippocampal tissues were extracted and single-cell suspensions prepared to determine the fluorescence intensity of Calcein AM using a Mitochondrial Permeability Transition Pore Assay Kit (Beyotime), according to the manufacturer’s instructions. Briefly, cells and fluorescence quenching solution were incubated in the dark at 37°C for 30 minutes. Subsequently, the fluorescence intensity of Calcein AM was measured at an emission of 517 nm with an excitation of 494 nm using a Flow Cytometer (Agilent Technologies, Inc., San Diego, CA, USA). Mean fluorescence intensity reflected the degree of mPTP opening, with fluorescence intensity negatively correlated with the degree of mPTP opening.

### Assessment of mitochondrial membrane potential

MMP (Δψm) was detected using the JC-1 MMP assay kit (Beyotime, C2006). Briefly, purified synaptic mitochondria (isolated from synaptosomes using a mitochondrial isolation kit [Biotronik, Berlin, Germany, C3606]) and JC-1 staining working solution were incubated at 37°C for 30 minutes. The fluorescence intensity of JC-1 monomers (λex 490 nm, λem 530 nm) and aggregates (λex 525 nm, λem 590 nm) were measured using a multi-functional microplate reader (Thermo Fisher Scientific Inc., Waltham, MA, USA). The results are represented as the ratio of red (aggregates)/green (monomers) fluorescence.

### Determination of adenosine triphosphate content

The ATP content of hippocampal synaptosomes was detected using the Enhanced ATP Assay Kit (Beyotime Biotechnology, S0027) according to the manufacturer’s instructions. Tissues were lysed in ATP lysis buffer, and after protein quantification, the protein in each sample was homogenized using lysis buffer. The ATP content was determined using a luminometer.

### Transmission electron microscopy

Hippocampal synaptosomes were fixed with 2.5% glutaraldehyde at 4°C overnight. After washing three times with PBS, the sections were fixed in 1% osmium tetroxide, stained with a 2% aqueous solution of uranyl acetate, and then dehydrated with gradients of different concentrations of ethanol and acetone. The samples were finally embedded in epoxy resin. Ultrathin sections (70 nm) were cut with an ultramicrotome, collected on a copper grid, and stained with 4% uranyl acetate and lead citrate. The ultrastructure of synaptosomes was imaged using a transmission electron microscope (Tecnai G2 Spirit Twin; FEI Company, Hillsboro, OR, USA).

### Golgi–Cox staining

Golgi staining was performed as previously described (Zhang et al., 2019). The FD Rapid Golgi Stain^TM^ Kit (FD Neuro Technologies, Columbia, MD, USA, PK401) was used in accordance with the manufacturer’s instructions. Hippocampal neurons were imaged using an auto microscope under Z-stack mode (20× object) for dendritic analysis. The dendrites from hippocampal neurons in the CA1 region were imaged using an Olympus BX53 microscope (63× oil object). Dendrite branches were traced using the NeuronJ plugin in ImageJ software, and the dendritic length calculated. Sholl analysis was applied to measure dendritic complexity. Dendritic spine density was detected along CA1 secondary dendrites starting from their point of origin on the primary dendrite.

### Hippocampal field potential recording

Mice were anesthetized with sevoflurane and decapitated. The brains were immediately removed and immersed in ice-cold dissection buffer containing 80 mM NaCl, 3.5 mM KCl, 4.5 mM MgSO_4_, 0.5 mM CaCl_2_, 1.25 mM NaH_2_PO_4_, 90 mM sucrose, 25 mM NaHCO_3_, and 10 mM glucose for 2–3 minutes. The brains were then cut using a vibratome into 300-μm thick sections in ice-cold dissection buffer continuously aerated with 95% O_2_ and 5% CO_2_. Sections were incubated in oxygenated artificial cerebrospinal fluid containing 126 mM NaCl, 2.5 mM KCl, 1.2 mM NaH_2_PO_4_, 1.2 mM MgSO_4_, 2.4 mM CaCl_2_, 26 mM NaHCO_3_, and 10 mM glucose (pH 7.4; continuously equilibrated with 95% O_2_ and 5% CO_2_) for 2 hours at 32°C. Hippocampal slices were transferred to the recording chamber and perfused with artificial cerebrospinal fluid at a rate of 2–3 mL/min at 32°C. Field excitatory post-synaptic potentials were evoked by inserting a concentric bipolar stimulating electrode into the Schaffer collateral/commissural afferents. Extracellular recording electrodes filled with artificial cerebrospinal fluid were inserted into the CA1 region. Baseline responses were collected every 30 seconds using an input stimulus intensity that induced 30%–40% of the maximum response. LTP was induced by three high-frequency stimuli (HFS: 100 Hz, duration 1 second, 10-minute intervals). Averaged field excitatory post-synaptic potential slopes over the last 20 minutes of recording were used for analysis.

### Statistical analysis

Statistical analyses were performed using GraphPad Prism (version 7.0.0 for Windows, GraphPad Software, Boston, MA, USA, www.graphpad.com). Clampfit 10.7 (Molecular Devices, San Jose, CA, USA) was used to analyze electrophysiological data. Figures were prepared using Adobe Photoshop 2020 (Adobe, San Jose, CA, USA). All data are expressed as mean ± standard error of mean (SEM). When data followed a normal distribution, two-tailed unpaired *t*-tests were used to compare parameters between two groups. One-way/two-way analysis of variance followed by Tukey’s *post hoc* analysis was used for multiple comparisons. In cases where equal variance assumptions were not met, a statistical significance was evaluated using the Mann–Whitney *U* test (between two groups) or Kruskal–Wallis test *post hoc* Dunn’s multiple comparison test (among three groups or more). Statistical significance was set at *P* < 0.05.

## Results

### Effects of anesthesia/surgery on cognitive function, synaptic mitochondria, and synaptic plasticity

**Figure 2:**
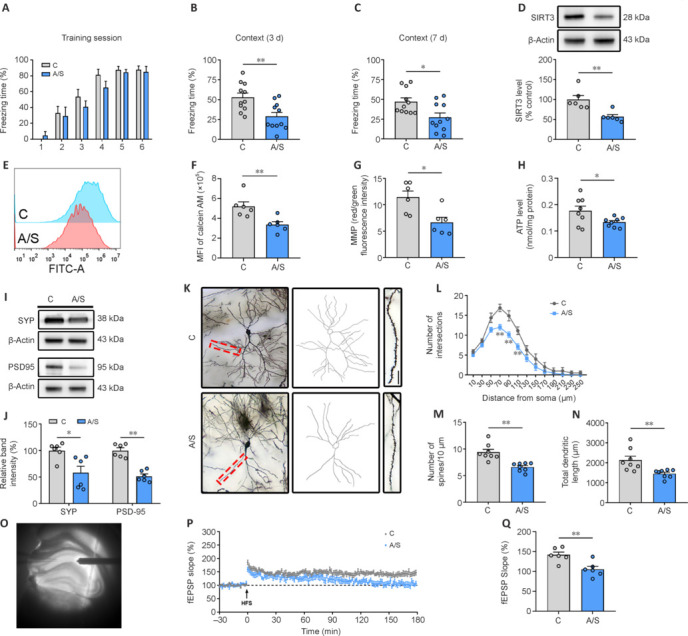
Cognitive function, and hippocampal synaptic mitochondrial function and synaptic plasticity are impaired after anesthesia and surgery in aged mice. (A) Percentage of freezing time during the FCT training session one day before anesthesia/surgery (*n* = 11 mice/group; two-way analysis of variance followed by *post hoc* Tukey’s test). (B, C) Percentage of freezing time in the context test (FCT) on days 3 (two-tailed unpaired Student’s *t*-test) and 7 (Mann–Whitney *U* test) post-anesthesia/surgery (*n* = 11 mice/group). (D) Representative blots and quantification of SIRT3 expression in hippocampal synaptosomes post-anesthesia/surgery (*n* = 6 mice/group; Mann–Whitney *U* test). SIRT3 expression levels were normalized to β-actin for each sample. (E, F) Opening of mPTP was examined using flow cytometry (*n* = 6 mice/group; two-tailed unpaired Student’s *t*-test). MFI was negatively correlated with mPTP opening. (G) MMP after anesthesia and surgery in control and anesthesia/surgery groups of aged mice (*n* = 6 mice/group; two-tailed unpaired Student’s *t*-test). (H) ATP levels after anesthesia and surgery in aged mice of control and anesthesia/surgery groups (*n* = 8 mice/group; two-tailed unpaired Student’s *t*-test). (I, J) Representative blots and quantification of SYP (Mann–Whitney *U* test) and PSD95 (two-tailed unpaired Student’s *t*-test) after anesthesia/surgery (*n* = 6 mice/group). (K) Representative images and camera tracings of Golgi–Cox staining for CA1 neurons and apical spines in hippocampal CA1 neurons. Scale bar: 10 µm. (L) Sholl analysis measured the dendritic complexity of reconstructed pyramidal neurons (*n* = 8 neurons/group; two-way analysis of variance followed by *post hoc* Tukey’s test). (M) Quantification of dendritic spine density (*n* = 8 neurons/group; two-tailed unpaired Student’s *t*-test). (N) Quantification of total dendritic length (*n* = 8 neurons/group; Mann–Whitney *U* test). (O) Sample image depicting the location of a stimulating electrode in the CA3 region and a recording electrode in the CA1 region. (P) HFS-induced LTP was recorded in the hippocampal CA1 region. The arrow indicates time of HFS application. (Q) Averaged fEPSP slope during the last 30 minutes (*n* = 6 mice/group; two-tailed unpaired Student’s *t*-test). Data are presented as mean ± SEM for each group. *P < 0.05, **P < 0.01, *vs*. control group. A/S: Anesthesia/surgery; C: control; fEPSP: field excitatory postsynaptic potential; HFS: high-frequency stimuli; MFI: mean fluorescence intensity; MMP: mitochondrial membrane potential; mPTP: mitochondrial permeability transition pore; PSD95: postsynaptic density 95; SIRT3: sirtuin 3; SYP: synaptophysin.

The OFT showed no statistically significant distinctions in the overall distance traveled by mice, both before and after surgery, between the control group and A/S group (**Additional Figure 1A–C**). The FCT demonstrated a comparable ability to acquire memory, with both the control and anesthesia/surgery mice reaching peak performance by the sixth training session (**Figure 2A**). During the testing phase, it became evident that mice exposed to anesthesia and surgery exhibited reduced freezing time in the context test on postoperative days 3 and 7, in comparison to control mice (**Figure 2B** and **C**). Nevertheless, no notable differences were observed between the two groups in the tone test (**Additional Figure 1D** and **E**). These data provide evidence that anesthesia and surgery can lead to postoperative cognitive impairment in aged mice.

Immunofluorescence staining was performed to investigate whether SIRT3 localization in the CA1 region was cell type-specific. Representative images showed that SIRT3 co-localized with neuronal nuclei (NeuN) but not GFAP- or Iba-1 immunopositivity (**Additional Figure 1F**). The negative control for SIRT3 immunocytochemistry is shown in **Additional Figure 1G**. Subsequently, we isolated hippocampal synaptosomes to examine SIRT3 protein expression. **Additional Figure 1H** shows the results of the discontinuous Percoll gradient synaptosome extraction procedure. Synaptosomes that were enriched at the 10/23% interface were visualized by transmission electron microscopy using low- and high-power objectives. Notably, this showed significant downregulation of SIRT3 protein expression following the anesthesia/surgery procedure in mouse synaptosomes (100.00% *vs*. 57.09%, *P* = 0.004) (**Figure 2D**). We also demonstrated increased mPTP opening (**Figure 2E** and **F**), reduced MMP (**Figure 2G**), and diminished ATP production (**Figure 2H**) in aged mice subjected to anesthesia and surgery, in comparison to control mice. These findings collectively suggest a marked impairment in synaptic mitochondrial function subsequent to the anesthesia and surgery procedure.

To explore the potential link between impaired synaptic mitochondrial function and downregulation of hippocampal synaptic plasticity, we examined the expression levels of two synaptic-related proteins: synaptophysin and PSD95 (**Figure 2I** and **J**). The findings showed notable reductions in both synaptophysin and PSD95 expression within hippocampal synaptosomes subsequent to anesthesia/surgery. Furthermore, **Figure 2K** presents representative morphology and camera tracings of CA1 neurons, with representative images of apical spines. The data indicated decreases in dendritic branch complexity, total length of dendrites, and density of dendritic spines in hippocampal neurons from the anesthesia/surgery group, compared with the control group (**Figure 2L** and **N**).

Electrophysiological studies showed a reduction in field excitatory post-synaptic potential slope during LTP among aged mice following anesthesia/surgery, compared with the control mice (**Figure 2O–Q**). These findings collectively point towards significant deficits in both the structural and functional synaptic plasticity of hippocampal neurons in aged mice subsequent to anesthesia/surgery.

### Sirtuin 3 overexpression in hippocampal synaptosomes ameliorates anesthesia/surgery-induced synaptic mitochondrial dysfunction and cognitive impairment

**Figure 3:**
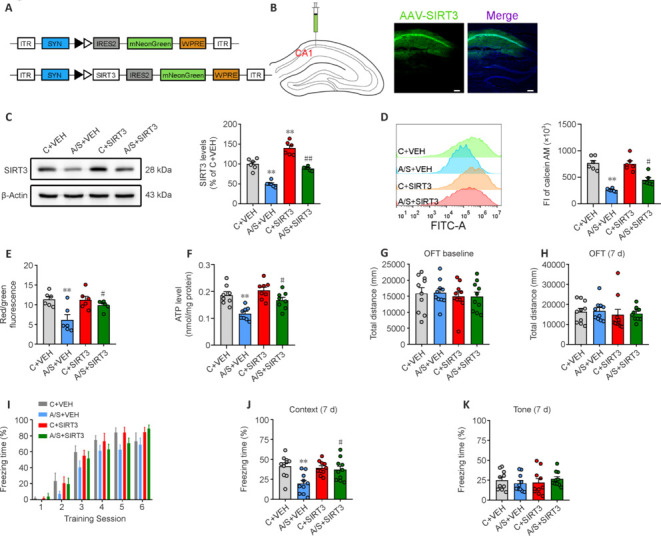
SIRT3 overexpression in synaptosomes alleviates synaptic mitochondrial dysfunction and cognitive impairment induced by anesthesia and surgery. (A) Location of viral microinjection for AAV-SIRT3 or control AAV-VEH. (B) Fluorescence images showing efficient expression of AAV-SIRT3 vector in the CA1 region. Scar bars: 100 µm. (C) Representative blots and quantification of SIRT3 expression in hippocampal synaptosomes after AAV-SIRT3 or AAV-VEH microinjection (*n* = 6 mice/group; one-way analysis of variance followed by *post hoc* Tukey’s test). (D–F) Changes in mPTP opening (*n* = 6 mice/group; Kruskal–Wallis test with *post hoc* Dunn’s multiple comparison test), MMP (*n* = 6 mice/group; one-way analysis of variance followed by *post hoc* Tukey’s test), and ATP levels (*n* = 8 mice/group; one-way analysis of variance followed by *post hoc* Tukey’s test) in hippocampal synaptic mitochondria after AAV-SIRT3 or AAV-VEH microinjection. (G, H) Total distance traveled during the OFT among the four groups 1 day before (*n* = 10 mice/group; one-way analysis of variance followed by *post hoc* Tukey’s test) and 7 days after anesthesia/surgery (*n* = 10 mice/group; Kruskal–Wallis test with *post hoc* Dunn’s multiple comparison test). (I–K) Percentage of freezing time during the training session 1 day before anesthesia/surgery (*n* = 10 mice/group; two-way analysis of variance followed by *post hoc* Tukey’s test), as well as in the context test and tone test 7 days after anesthesia/surgery (*n* = 10 mice/group; one-way analysis of variance followed by *post hoc* Tukey’s test). Data are presented as mean ± SEM for each group. **P < 0.01, *vs*. C + VEH group; #*P* < 0.05, ##*P* < 0.01, *vs*. A/S + VEH group. A/S: Anesthesia/surgery; AAV-SIRT3: rAAV-SYN-pre-mGRASP-SIRT3-IRES2-mNeonGreen vector; AAV-VEH: rAAV-SYN-pre-mGRASP-MCS-IRES2-mNeonGreen vehicle; C: control; OFT: open-field test; SIRT3: sirtuin 3; VEH: vehicle.

**Figure 4:**
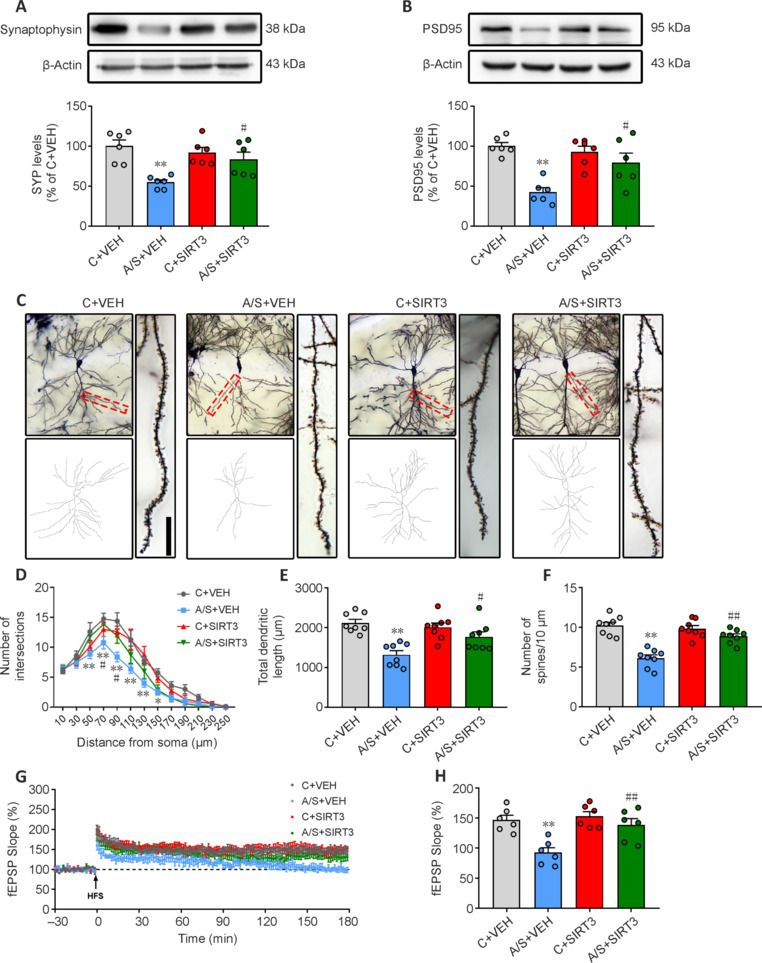
SIRT3 overexpression in synaptosomes ameliorates the decline in dysfunctional hippocampal synaptic plasticity induced by anesthesia and surgery. (A, B) Representative blots and quantification of synaptophysin and PSD95 in hippocampal synaptosomes (*n* = 6 mice/group; one-way analysis of variance followed by *post hoc* Tukey’s test). (C) Representative images of Golgi–Cox staining in CA1 neurons. Scale bar: 10 µm. (D) Sholl analysis measured the dendritic complexity of dendritic intersections (*n* = 8 neurons/group; two-way analysis of variance followed by *post hoc* Tukey’s test). (E) Quantification of total dendritic length (*n* = 8 neurons/group; one-way analysis of variance followed by *post hoc* Tukey’s test). (F) Quantification of dendritic spine density (*n* = 8 neurons/group; one-way analysis of variance followed by *post hoc* Tukey’s test). (G) HFS-induced LTP was recorded in the hippocampal CA1 region. (H) Averaged fEPSP slope during the last 30 minutes (*n* = 6 mice/group; one-way analysis of variance followed by *post hoc* Tukey’s test). Data are presented as mean ± SEM for each group. *P < 0.05, **P < 0.01, *vs.* C + VEH group; #*P* < 0.05, ##*P* < 0.01, *vs*. A/S + VEH group. A/S: Anesthesia/surgery; C: control; fEPSP: field excitatory postsynaptic potential; HFS: high-frequency stimuli; LTP: long-term potentiation; PSD95: postsynaptic density 95; VEH: vehicle.

Injections of AAV-SYN-SIRT3-mNeonGreen or AAV-SYN-mNeonGreen were administered into the hippocampal CA1 region. Placement accuracy and SIRT3 expression were subsequently verified (**Figure 3A** and **B**). Following AAV-SIRT3 injection, substantial augmentation of SIRT3 protein levels was observed within the hippocampus of the anesthesia/surgery group, demonstrating successful transfection of the viral vector (**Figure 3C**). This increased SIRT3 expression effectively counteracted the anesthesia/surgery-induced increase in mPTP opening, decrease in MMP, and reduction in ATP production within synaptic mitochondria (**Figure 3D–F**). Overexpression of SIRT3 did not lead to significant alterations in the locomotor capacity of mice (**Figure 3G** and **H**) or freezing time during FCT training (**Figure 3I**). However, it did significantly alleviate the cognitive impairment triggered by anesthesia/surgery in the context test (**Figure 3J**), while no substantial variance was detected in the tone test (**Figure 3K**). These findings underscore the potential pivotal role of SIRT3 in the emergence of anesthesia/surgery-induced cognitive impairment, particularly through regulation of synaptic mitochondrial energy provision. Furthermore, the increased expression of SIRT3 had a mitigating effect on the anesthesia/surgery-induced reduction in synaptophysin and PSD95 expression (**Figure 4A** and **B**), decline in synaptic structural plasticity (**Figure 4C–F**), and impairment in LTP (**Figure 4G** and **H**) within aged mice. Collectively, these results suggest that enhanced SIRT3 expression within synaptosomes might act to ameliorate the dysfunctional synaptic plasticity induced by anesthesia and surgery in aged mice.

### SIRT3 binds and deacetylates CypD at K166

**Figure 5:**
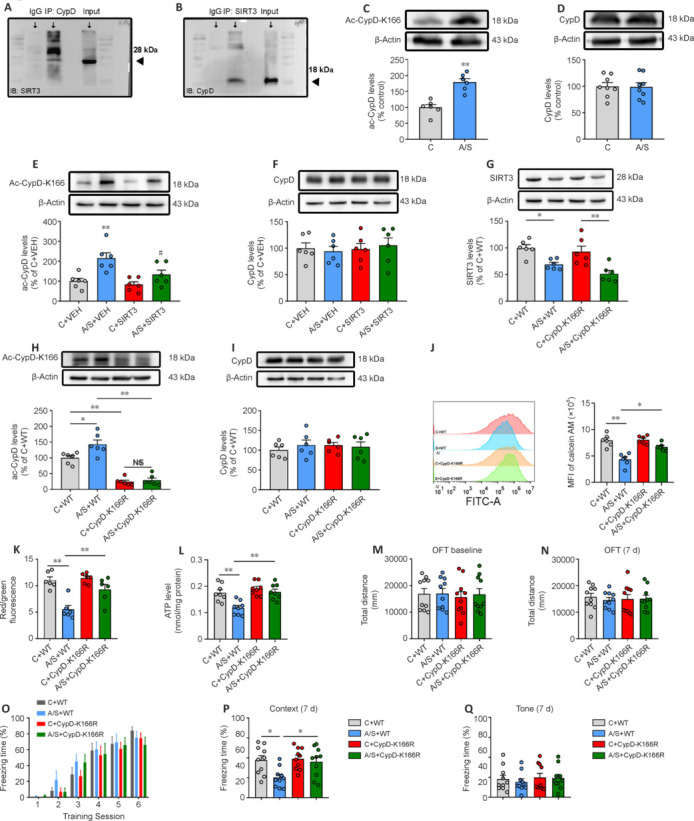
SIRT3 mediates deacetylation of CypD at the K166 site and substitution of lysine 166 to arginine in CypD (CypD-K166R) significantly reverses synaptic mitochondrial dysfunction and postoperative cognitive impairment in aged mice. (A, B) Interaction between SIRT3 and CypD was examined using SIRT3 or CypD antibodies and co-immunoprecipitation. (C, D) Changes of Ac-CypD-K166 and CypD expression in hippocampal synaptosomes 7 days after anesthesia/surgery (*n* = 6 mice/group; two-tailed unpaired Student’s *t*-test). (E, F) Representative blots and quantification of Ac-CypD-K166 and CypD in hippocampal synaptosomes after AAV-SIRT3 or AAV-VEH microinjection (*n* = 6 mice/group; one-way analysis of variance followed by *post hoc* Tukey’s test). **P < 0.01, *vs*. C + VEH group; #*P* < 0.05, *vs*. A/S + VEH group. (G–I) Representative western blots and quantification of SIRT3, Ac-CypD-K166, and CypD expression (*n* = 6 mice /group; one-way analysis of variance followed by *post hoc* Tukey’s test). (J–L) Changes in mPTP opening (*n* = 6 mice/group; one-way analysis of variance followed by *post hoc* Tukey’s test), MMP (*n* = 6 mice/group; Kruskal–Wallis test with *post hoc* Dunn’s multiple comparison test), and ATP levels (*n* = 8 mice/group; one-way analysis of variance followed by *post hoc* Tukey’s test). (M, N) Total distance traveled during the OFT 1 day before (Kruskal–Wallis test with *post hoc* Dunn’s multiple comparison test) and 7 days (one-way analysis of variance followed by *post hoc* Tukey’s test) after anesthesia/surgery (*n* = 10 mice/group). (O–Q) Percentage of freezing time during the training session 1 day before anesthesia/surgery (two-way analysis of variance followed by *post hoc* Tukey’s test), as well as in the context (one-way analysis of variance followed by *post hoc* Tukey’s test) and tone (Kruskal–Wallis test with *post hoc* Dunn’s multiple comparison test) tests 7 days after anesthesia/surgery (*n* = 10 mice/group). Data are presented as mean ± SEM for each group. *P < 0.05, **P < 0.01. A/S: Anesthesia/surgery; AAV-SIRT3: rAAV-SYN-pre-mGRASP-SIRT3-IRES2-mNeonGreen vector; AAV-VEH: rAAV-SYN-pre-mGRASP-MCS-IRES2-mNeonGreen vehicle; ATP: adenosine triphosphate; C: control; MMP: mitochondrial membrane potential; mPTP: mitochondrial permeability transition pore; SIRT3: sirtuin 3; VEH: vehicle.

Next, we examined the interaction between SIRT3 and CypD using co-immunoprecipitation (**Figure 5A** and **B**). This showed visible enhancements in the expression of Ac-CypD-K166 (**Figure 5C**), but not CypD (**Figure 5D**), within hippocampal synaptosomes following anesthesia/surgery. There was a significant reduction in the expression of Ac-CypD-K166 in mice overexpressing SIRT3, while the total levels of CypD remained unaffected (**Figure 5E** and **F**). These findings suggest that SIRT3 may facilitate deacetylation of CypD at the K166 site in aged mice subsequent to anesthesia/surgery.

### CypD-K166R mutation significantly ameliorates postoperative cognitive decline in aged mice

**Figure 6:**
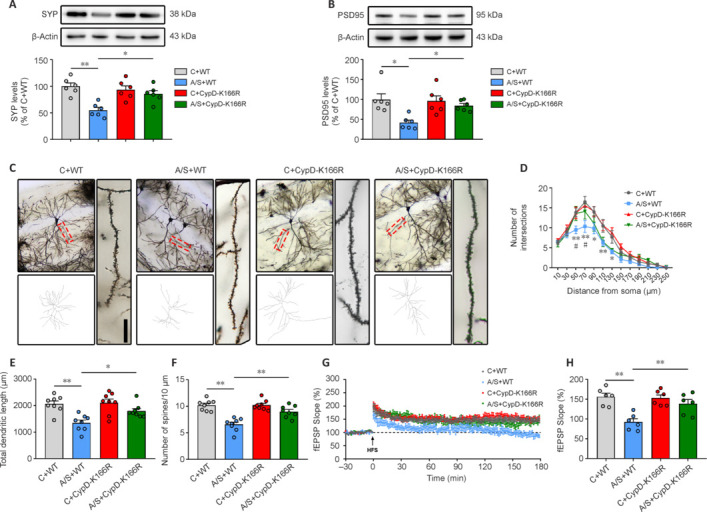
CypD-K166R substitution rescues surgery-induced hippocampal synaptic plasticity dysfunction in aged mice. (A, B) Representative blots and quantification of SYP (*n* = 6 mice/group; one-way analysis of variance followed by *post hoc* Tukey’s test) and PSD95 (*n* = 6 mice/group; Kruskal–Wallis test with *post hoc* Dunn’s multiple comparison test). (C) Representative images of Golgi–Cox staining of CA1 neurons. Scale bar: 10 µm. (D) Sholl analysis measured the dendritic complexity of dendritic intersections (*n* = 8 neurons/group; two-way analysis of variance followed by *post hoc* Tukey’s test). (E) Quantification of total dendritic length (*n* = 8 neurons/group; one-way analysis of variance followed by *post hoc* Tukey’s test). (F) Quantification of dendritic spine density (*n* = 8 neurons/group; one-way analysis of variance followed by *post hoc* Tukey’s test). (G) HFS-induced LTP was recorded in the hippocampal CA1 region. (H) Averaged fEPSP slope during the last 30 minutes among different groups (*n* = 6 mice/group; one-way analysis of variance followed by *post hoc* Tukey’s test). Data are presented as mean ± SEM for each group. *P < 0.05, **P < 0.01. A/S: Anesthesia/surgery; C: control; fEPSP: field excitatory postsynaptic potential; HFS: high-frequency stimuli; LTP: long-term potentiation; PSD95: postsynaptic density 95; SYP: synaptophysin; WT: wide type.

To understand the role of CypD-K166 acetylation in anesthesia/surgery-induced cognitive dysfunction, we examined CypD-K166R mutant mice. SIRT3 levels post-anesthesia/surgery remained stable in CypD-K166R mutants compared with wild-type mice (**Figure 5G**). Remarkably, CypD-K166R mice exhibited reduced CypD-K166 levels in control and anesthesia/surgery groups (**Figure 5H**), while the total levels of CypD remained unaffected (**Figure 5I**). Mitochondrial function tests showed that the CypD-K166R mutation mitigated anesthesia/surgery-induced effects on mPTP opening, MMP, and ATP production (**Figure 5J–L**). Behavioral assessments indicated unchanged locomotor activity and freezing times (**Figure 5M–O**). Notably, the CypD-K166R mutation improved anesthesia/surgery-induced cognitive impairment in the context test (**Figure 5P**) while not affecting the tone test (**Figure 5Q**). These findings highlight the significance of CypD-K166 acetylation in dNCR development. Further investigation revealed that CypD-K166R mutants exhibited increased synaptophysin and PSD95 expression post-anesthesia/surgery (**Figure 6A** and **B**). Golgi staining illustrated improved anesthesia/surgery-induced synaptic structural plasticity decline (**Figure 6C–F**). Moreover, the CypD-K166R mutation mitigated anesthesia/surgery-induced LTP impairment (**Figure 6G** and **H**).

### Inhibiting mitochondrial permeability transition pore opening attenuates anesthesia/surgery-induced synaptic mitochondrial dysfunction and cognitive impairment

**Figure 7:**
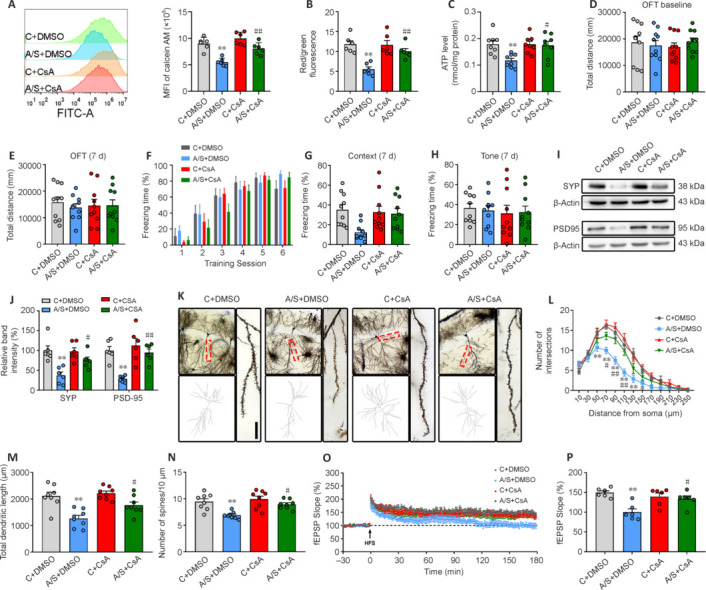
Inhibition of mPTP opening ameliorates anesthesia/surgery-induced synaptic mitochondrial dysfunction, cognitive impairment, and hippocampal synaptic plasticity dysfunction in aged mice. (A–C) Opening of mPTP, MMP, and ATP levels in synaptic mitochondria after CsA or DMSO injection in aged mice (*n* = 6–8 mice/group; one-way analysis of variance followed by *post hoc* Tukey’s test). (D, E) Total distance traveled during the OFT 1 day before and 7 days after anesthesia/surgery (*n* = 10 mice/group; one-way analysis of variance followed by *post hoc* Tukey’s test). (F–H) Percentage of freezing time during the training session at 1 day before anesthesia/surgery (two-way analysis of variance followed by *post hoc* Tukey’s test), as well as in the context (Kruskal–Wallis test with *post hoc* Dunn’s multiple comparison test) and tone (one-way analysis of variance followed by *post hoc* Tukey’s test) tests at 7 days after anesthesia/surgery (*n* = 10 mice/group). (I, J) Representative blots and quantification of SYP and PSD95 (*n* = 6 mice/group; one-way analysis of variance followed by *post hoc* Tukey’s test). (K) Representative images of Golgi–Cox staining of CA1 neurons. Scale bar: 10 µm. (L) Sholl analysis measured the dendritic complexity of dendritic intersections (*n* = 8 neurons/group; two-way analysis of variance followed by *post hoc* Tukey’s test). (M) Quantification of total dendritic length (*n* = 8 neurons/group; one-way analysis of variance followed by *post hoc* Tukey’s test). (N) Quantification of dendritic spine density (*n* = 8 neurons/group; one-way analysis of variance followed by *post hoc* Tukey’s test). (O) HFS-induced LTP was recorded in the hippocampal CA1 region. (P) Averaged fEPSP slope during the last 30 minutes (*n* = 6 mice/group; one-way analysis of variance followed by *post hoc* Tukey’s test). Data are presented as mean ± SEM for each group. *P < 0.05, **P < 0.01, *vs*. C + DMSO group; #*P* < 0.05, ##*P* < 0.01, *vs.* A/S + DMSO group. A/S: Anesthesia/surgery; ATP: adenosine triphosphate; C: control; CsA: cyclosporine A; DMSO: dimethyl sulfoxide; fEPSP: field excitatory postsynaptic potential; HFS: high-frequency stimuli; LTP: long-term potentiation; MMP: mitochondrial membrane potential; mPTP: mitochondrial permeability transition pore; SYP: synaptophysin.

Finally, CsA administration caused significant reductions in mPTP opening, along with elevated MMP and ATP levels in synaptic mitochondria of aged mice subjected to anesthesia and surgery (**Figure 7A–C**). Furthermore, CsA effectively ameliorated cognitive impairment in the anesthesia/surgery group without exerting any influence on locomotor activity (**Figure 7D–H**). Moreover, CsA reversed dysfunctional hippocampal synaptic plasticity in the anesthesia/surgery group (**Figure 7I** and **J**). Additionally, CsA improved hippocampal synaptic structure (**Figure 7K–N**) and functional plasticity (**Figure 7O** and **P**). These findings suggest that inhibiting mPTP opening can effectively mitigate the decline in synaptic mitochondrial function and cognitive impairment induced by anesthesia and surgery in aged mice.

## Discussion

Perioperative neurocognitive disorders, comprising postoperative delirium, dNCR, and postoperative neurocognitive disorder, have been identified before or after surgery. dNCR specifically pertains to the cognitive impairment experienced by patients within thirty days following anesthesia/surgery.

The precise mechanisms contributing to the development of dNCR are not yet fully understood. Recent investigations have brought attention to the critical role of the deacetylase enzyme, SIRT3, in contributing to the mitochondrial dysfunction associated with oxidative stress in neurodegenerative disorders like Parkinson’s disease and AD (Shen et al., 2020; Zhou and Tan, 2020; Cartas-Cejudo et al., 2023). Moreover, studies indicate that elevating SIRT3 levels suppresses the mitochondrial oxidative stress response and prolongs the lifespan of primary cultured hippocampal neurons (Cheng et al., 2016). Nonetheless, the exact involvement of SIRT3 in the pathogenesis of dNCR remains to be elucidated.

In the present study, we found that anesthesia/surgery caused cognitive impairment in aged mice at 3 and 7 days after anesthesia/surgery, decreased SIRT3 expression, and increased Ac-CypD-K166 expression in hippocampal synaptosomes of mice. Overexpression of SIRT3 in CA1 synaptosomes attenuated anesthesia/surgery-induced cognitive impairment, along with acetylation of CypD at K166. Further studies using CypD-K166R mutant mice demonstrated amelioration of anesthesia/surgery-induced synaptic mitochondrial dysfunction and cognitive impairment. Furthermore, inhibiting mPTP opening by CsA attenuated the decline in synaptic mitochondrial function and cognitive impairment induced by anesthesia/surgery in aged mice. Other studies have also demonstrated that SIRT3 might be involved in the mechanism of perioperative neurocognitive disorder in aged mice (Liu et al., 2021; Dai et al., 2024). Further, that blocking the mPTP channel might attenuate sevoflurane-induced perioperative neurocognitive disorder (Zhang et al., 2012; Yang et al., 2024), but the specific molecular SIRT3 substrate that regulates the mPTP was unknown. These results support the hypothesis that SIRT3-mediated CypD deacetylation regulates mPTP opening, and may serve at least partially, as the mechanism of postoperative cognitive decline via synaptic mitochondrial function in aged mice.

Synaptic plasticity is the cell model of learning and memory (Yang et al., 2025). Synaptic activity plays a pivotal role in cognitive processes and demands substantial energy provision. Synaptic mitochondria gather at presynaptic membrane locations to furnish the requisite energy for synaptic function. Given the elevated energy requirements of synapses, synaptic mitochondria are more susceptible to oxidative stress compared with their non-synaptic counterparts. While mitochondrial oxidative phosphorylation accounts for over 95% of intracellular reactive oxygen species (ROS) generation, mitochondria typically possess the capacity to effectively manage excess ROS production and sustain a dynamic equilibrium under normal conditions (Xie et al., 2015; Zou et al., 2015). However, in pathological scenarios, the accumulation of ROS beyond acceptable levels impairs mitochondrial function, culminating in opening of the mPTP. This consequently leads to the release of substantial quantities of free radicals, cytochrome C, and activated caspase-3 into the cytoplasm, ultimately triggering apoptosis (Korge et al., 2015). In prior research, we demonstrated that SIRT3-mediated deacetylation of CypD restrains ROS generation, enhances mitochondrial performance, and mitigates neuropathic pain (Yan et al., 2022). In the context of the present study, we found diminished synaptic MMP and reduced ATP synthesis in the hippocampus of mice following surgery. However, interventions involving the upregulation of SIRT3, downregulation of CypD acetylation at the K166 site, and administration of the mPTP-blocking agent, CsA, all led to substantial enhancements in synaptic MMP levels and restoration of ATP production. These findings collectively suggest that SIRT3-mediated deacetylation of CypD could play a pivotal role in modulating the energy supply of synaptic mitochondria, thereby potentially influencing the development of dNCR.

Neuronal synaptic plasticity stands as a pivotal factor in cognitive function (Briz et al., 2017; Chiu et al., 2017; Notaras et al., 2020). In mice with AD, cognitive deterioration has been linked to compromised dendritic spine density within the hippocampus and inhibition of LTP (Lourenco et al., 2019; Cangalaya et al., 2023). Furthermore, both anesthesia and surgical procedures have been associated with a loss of hippocampal dendritic spines and subsequent apoptosis, which reportedly contributes to cognitive decline in aged mice (Qiu et al., 2020). LTP is a phenomenon denoting the heightened efficiency of synapses after high-frequency excitation of synaptic transmission, and serves as a molecular model for understanding learning and memory (Penn et al., 2017; Kulik et al., 2019). It is intricately involved in the localized restructuring of dendritic spines and synapses (Bourne and Harris, 2012; Watson et al., 2016). In our present study, we noted impaired structural and functional synaptic plasticity in the CA1 region of the hippocampus in aged mice after surgery. This was demonstrated by a notable reduction in the number of dendritic branches, overall dendritic length, dendritic spine density, and impaired LTP induction. However, interventions such as the augmentation of synaptosomal SIRT3, alteration of the CypD-K166 acetylation site, and administration of the mPTP inhibitor, CsA, were found to reverse these detrimental alterations in synaptic plasticity. This compellingly suggests a plausible link between SIRT3-mediated CypD deacetylation and the modulation of synaptic plasticity, thereby potentially influencing cognitive behaviors.

Despite the valuable insights gained from this study, several limitations should be acknowledged. First, our focus was on postoperative dNCR in aged mice, and further investigation into the long-term postoperative cognitive function changes is warranted owing to the broad time span of postoperative cognitive impairment. Second, sex, anesthesia scheme, and surgical procedure might influence the diversity of postoperative cognitive dysfunction, and these factors need to be individually investigated. Third, although the FCT and OFT results in mice provide valuable insights, further validation through clinical studies are necessary to correlate these findings with human cognitive function and behavior. Fourth, studies have shown that changes in SIRT3 levels alter metabolism (Wang et al., 2019; Qian et al., 2023). Therefore, we analyzed the body weight of mice among groups at one day before surgery and 7 days after surgery and found no significant differences (**Additional Figure 2**). The reason may be that anesthesia and surgery were relatively acute and short-term periods, but we still cannot exclude the potential metabolic influence of SIRT3 in dNCR of aged mice and further study is needed. Lastly, while our findings emphasize the importance of SIRT3 in regulating dNCR, the involvement of other SIRT family members in the occurrence of dNCR remains to be determined.

In summary, the outcomes of this study underscore the potential importance of disturbed energy provision in synaptic mitochondria as a contributing factor to the mechanisms underlying dNCR. Involvement of mPTP in compromised mitochondrial energy delivery emerges as a pivotal aspect of this process. Notably, modulation of mPTP via SIRT3-mediated deacetylation of CypD emerges as a potential central control point. These findings provide invaluable insights into the nexus between synaptic energy supply, synaptic plasticity, and cognitive function. This in turn encourages further investigations to understand the pathogenesis of perioperative neurocognitive disorders, and subsequently, devise targeted interventions for their amelioration.

## Additional files:

***Additional Figure 1:*** *Anesthesia and surgery do not impair locomotor activity and hippocampus-independent learning and memory in aged mice.*

Additional Figure 1 Anesthesia and surgery do not impair locomotor activity and hippocampal-independent learning and memory in aged mice.
(A–C) Total distance traveled by the mice atone day before (two-tailed unpaired Student’s *t*-test) and 3 (Mann- Whitney *U* test) and 7 days (two-tailed unpaired Student’s *t*-test) after anesthesia/surgery during the OFT (n = 11 mice/group). (D, E) Percentage of freezing time in the tone test on days 3 (Mann-Whitney *U* test) and 7 (twotailed unpaired Student’s *t*-test) post-anesthesia/surgery (n = 11 mice/group). (F) Representative images with double immunofluorescence staining for SIRT3 (green, Alexa 488) and neuronal nuclei (NeuN, red, Alexa 594), GFAP (red, Alexa 594), and Iba-1 (red, Alexa 594). Scale bars: 100 μm. (G) The negative control for SIRT3 immunocytochemistry (green, Alexa 488). Scale bars: 100 μm. (H) Images of the synaptosome (arrows) extraction procedure and synaptosomes visualized by transmission electron microscopy using low- and high-power objectives. Data are presented as mean ± SEM for each group. A/S: Anesthesia/surgery; C: control; DAPI: 4',6- diamidino-2-phenylindole; GFAP: glial fibrillary acidic protein; Iba-1: ionized calcium-binding adapter molecule 1; OFT: open-field test; PBS: phosphate buffer saline; SIRT3: sirtuin 3.

***Additional Figure 2:*** *Body weight of mice among groups at 1 day before surgery and 7 days after surgery.*

Additional Figure 2 Body weight of mice among groups at 1 day before surgery and 7 days after surgery.
(A–G) Change of body weight between pre- and post-surgery in A/S (A), A/S + VEH (B), A/S + SIRT3 (C), A/S + WT (D), A/S + CypD-K166R (E), A/S + DMSO (F), and A/S + CsA (G) groups for aged mice. A/S: Anesthesia/surgery; CsA: cyclosporine A; DMSO: dimethyl sulfoxide; n.s: no significance; SIRT3: sirtuin 3; VEH: vehicle; WT: wide type.

## Data Availability

*All data relevant to the study are included in the article or uploaded as Additional files*.
