# Supplementary figures and images for: Mechanism of action of synaptic mitochondrial damage in delayed cognitive recovery
Source: Neural Regen Res. 2025 Mar 25;21(6):2457–66. doi: 10.4103/NRR.NRR-D-24-01126 (PMC13211829; doi:10.4103/NRR.NRR-D-24-01126)

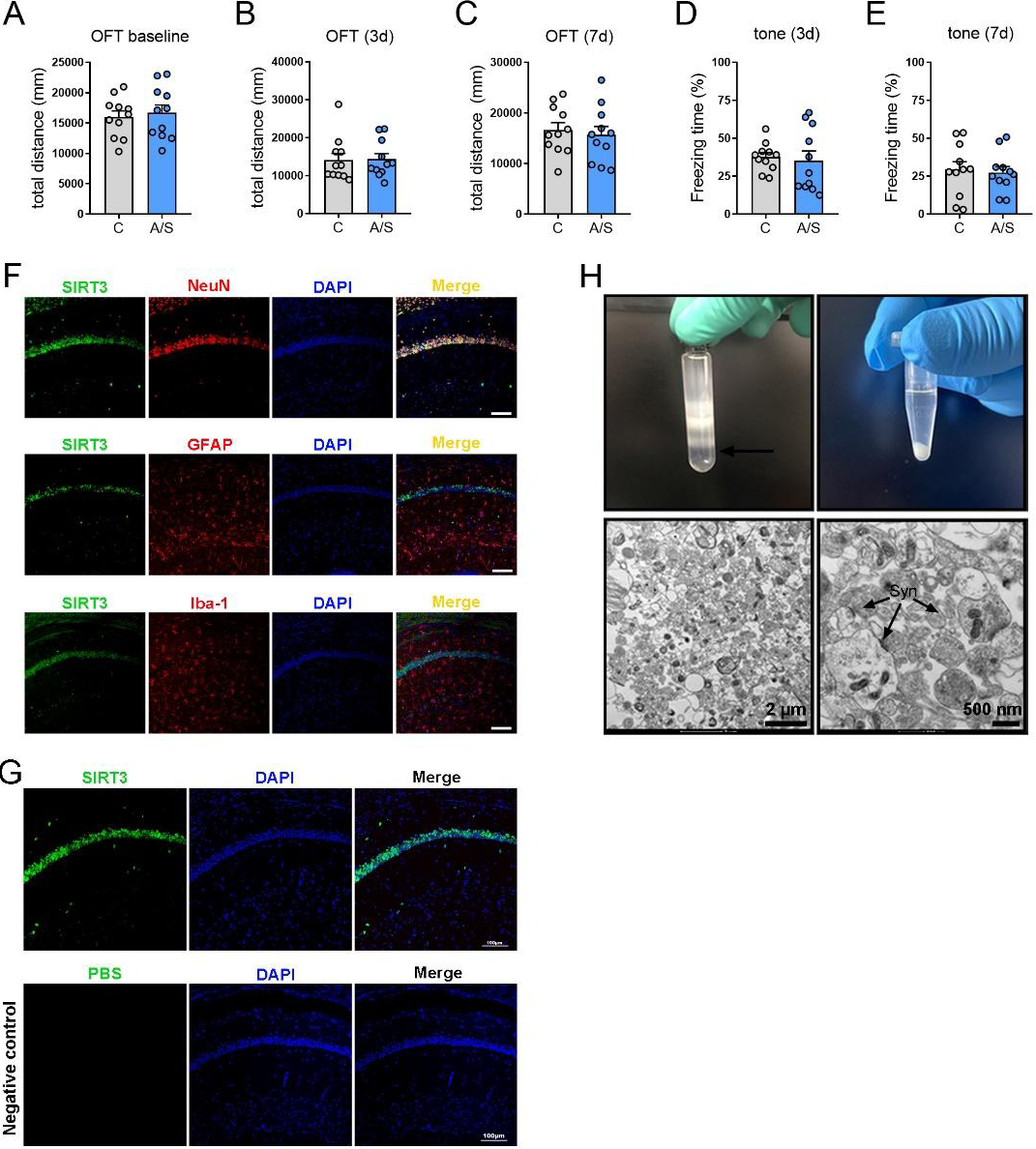

Supplement: Supplementary file 1 [file NRR-21-2457_Suppl1.tif]

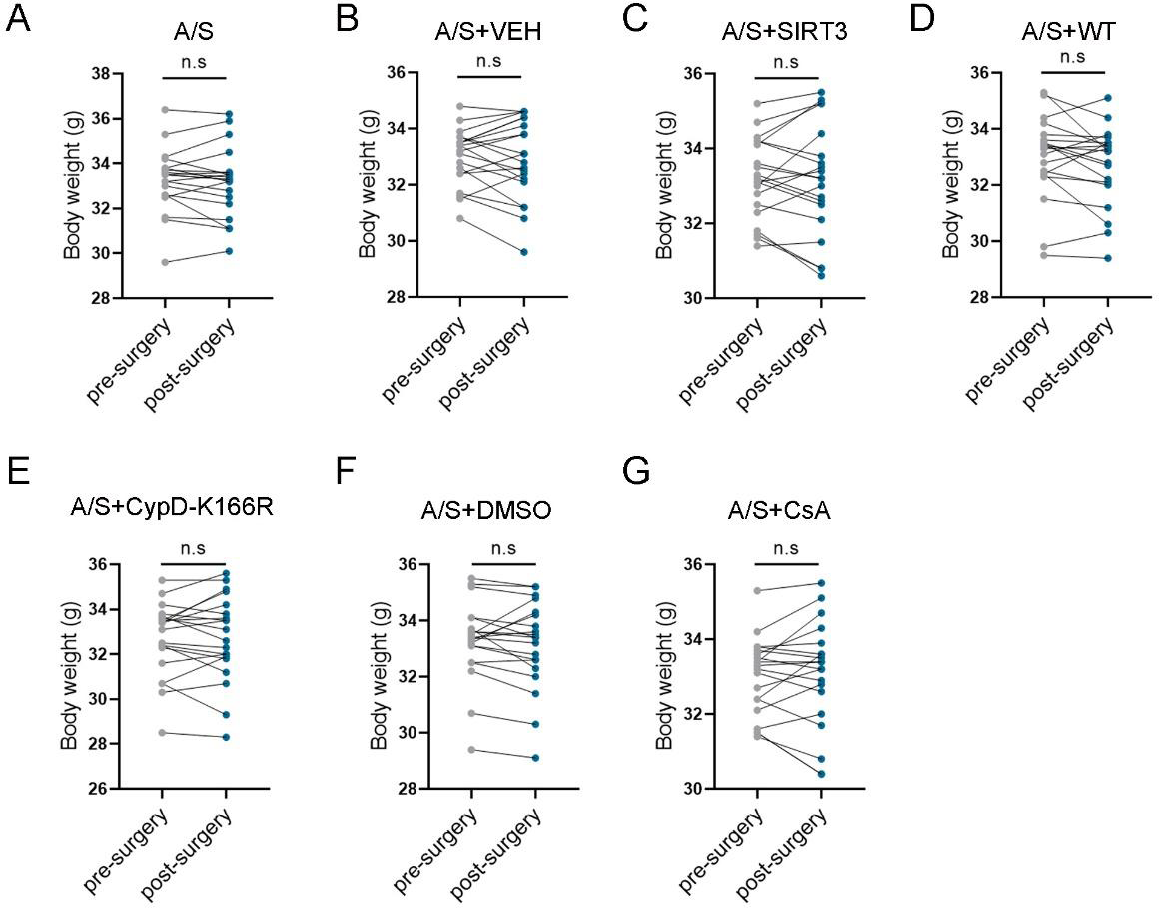

Supplement: Supplementary file 2 [file NRR-21-2457_Suppl2.tif]
